# Early Discharge of Very Preterm Infants with Home Nasogastric Tube Feeding Is Not Associated with Increased Parental HADS Anxiety or Depression Scores

**DOI:** 10.3390/nu18162728

**Published:** 2026-08-20

**Authors:** Rahel Schuler, Lea Woitschitzky, Burkhard Brosig, Beate Priewasser, Edda Hofstätter, Harald Ehrhardt, Walter A. Mihatsch

**Affiliations:** 1 Division of Neonatology, Department of Pediatrics and Adolescent Medicine, Paracelsus Medical University, 5020 Salzburg, Austria; e.hofstaetter@salk.at; 2 Family Psychosomatics, Department of Pediatrics and Neonatology, Justus-Liebig-University Giessen, 35392 Giessen, Germany; lea.woitschitzky@uk-gm.de (L.W.); burhard.brosig@uk-gm.de (B.B.); 3 Institute for Early Life Care, Paracelsus Medical University, 5020 Salzburg, Austria; beate.priewasser@pmu.ac.at; 4 Department of Neonatology, Charité—Universitätsmedizin Berlin, 13344 Berlin, Germany; harald.ehrhardt@charite.de; 5 Division of Neonatology and Pediatric Intensive Care Medicine, Department of Pediatrics and Adolescent Medicine, University Medical Center Ulm, Eythstr. 24, 89075 Ulm, Germany; walter.mihatsch@uni-ulm.de; 6 Department of Pediatrics, Balingen Hospital, 72336 Balingen, Germany

**Keywords:** very preterm infants, early discharge, home nasogastric tube feeding, maternal and paternal depression and anxiety

## Abstract

**Background:** Early discharge with home nasogastric (NGT) feeding has been associated with favorable infant outcomes and high parental satisfaction. However, its impact on parental mental health has been insufficiently studied. We aimed to evaluate symptoms of anxiety and depression in mothers and fathers of very preterm infants discharged with or without NGT feeding. **Methods:** This prospective cohort study was conducted at a German tertiary perinatal center between October 2020 and May 2024. A total of 238 preterm infants with a birth weight ≤ 1500 g and/or a postmenstrual age (PMA) ≤ 32 weeks were included; 131 were discharged with full oral feeding and 107 with NGT feeding. Symptoms of anxiety and depression were assessed in mothers and fathers using the Hospital Anxiety and Depression Scale (HADS-D) at discharge and at three months corrected age (CA). A total of 201 parent–infant dyads were included. HADS-D assessments were completed by 155 mothers and 135 fathers at discharge and by 121 mothers and 119 fathers at three months CA. **Results:** At discharge, depression and anxiety scores did not differ between parents of infants discharged with and without NGT feeding. At three months CA, mothers of infants discharged on full oral feeding had higher depression and anxiety scores than mothers of infants discharged with NGT feeding (4.95 ± 3.62 vs. 3.18 ± 2.89; *p* ≤ 0.01 and 6.42 ± 3.85 vs. 4.79 ± 3.72; *p* = 0.04), and clinically significant depression scores (≥8) were more frequent (23.53% vs. 10.45%; *p* = 0.04). Similarly, fathers in the non-tube-feeding group had higher depression scores at 3 months CA (4.78 ± 3.74 vs. 3.48 ± 2.72; *p* = 0.04), with a trend towards a greater proportion showing clinically significant depression scores (24.14% vs. 13.11%; *p* = 0.12. **Conclusions:** Early discharge of very preterm infants with home NGT feeding was not associated with increased parental psychological distress at discharge and at 3 months CA. Across the outcomes, parental distress tended to be lower at 3 months CA with NGT feeding at discharge. However, as no NGT × time interaction was statistically significant after correction for multiple testing, these group differences should be interpreted as exploratory in nature.

## 1. Introduction

In most neonatal intensive care units (NICUs), discharge of preterm infants is contingent upon the establishment of full oral feeding [1]. This practice may delay discharge until the expected date of delivery or beyond [2]. Prolonged hospitalization has been associated with adverse neurodevelopmental outcomes in preterm infants [3] and is a significant source of stress for parents. In particular, the final weeks of hospitalization—until removal of the feeding tube is achieved—are often experienced as especially burdensome [4]. As an alternative to prolonged hospitalization during the final weeks prior to discharge, early discharge programs incorporating home nasogastric tube (NGT) feeding have been implemented. These programs vary in structure, with some relying on telemedicine-based monitoring [4,5], while others are supported by neonatal homecare nursing visits and outpatient follow-up [6,7]. Early discharge programs incorporating home NGT feeding have been evaluated with respect to infant outcomes, parental experience, and healthcare utilization, with no evidence of negative effects [6,7]. Specifically, breastfeeding rates were higher in some studies and not reduced in any [8,9,10]. Infant growth through three months CA remained adequate, and readmission rates were not increased [11]. Parental satisfaction with early discharge was consistently high [7,12,13]. Furthermore, early discharge with home NGT feeding has the potential to reduce healthcare costs [11,13,14].

While one previous study has suggested that early discharge may not adversely affect parental mental health among moderately preterm infants [9], the psychological impact of early discharge with ongoing tube feeding on parents of very preterm infants remains largely unexplored. The increased caregiving responsibilities associated with home NGT feeding, combined with the ongoing medical vulnerability of very preterm infants, may increase parental stress and uncertainty. Therefore, discharge at a lower postmenstrual age (PMA) with continued medical care at home may represent a challenging transition for families and could increase parental anxiety in this high-risk population. Further characterization of parental mental health outcomes is therefore essential, as parental depression and anxiety symptoms have been linked to adverse neurodevelopmental and behavioral outcomes in children [15,16]. Notably, maternal depressive symptoms have been linked to deficits in social functioning, behavior, and executive functioning in preterm children at school age, whereas corresponding associations have not been consistently observed for fathers [17].

Therefore, the aim of the present study was to assess symptoms of anxiety and depression in mothers and fathers of very preterm infants at discharge and again at three months CA. In addition, we evaluated parents’ perceived readiness for discharge and feeding-related stress at home four weeks after discharge. Outcomes were compared between families discharged with and without NGT feeding.

## 2. Materials and Methods

### 2.1. Study Design

This study was embedded within a prospective longitudinal cohort study conducted at a German tertiary perinatal center investigating the impact of family-centered care (FCC). Family-centered care was progressively implemented throughout the study period and was available to all study participants. In addition, psychological support was routinely offered to all parents throughout their infant’s hospital stay. The study is registered at ClinicalTrials.gov (NCT05286983) and was approved by the local Institutional Review Board prior to initiation (AZ 153/20). Written informed consent was obtained from both caregivers within the first two weeks after birth.

The primary outcome of the overall study was the PMA at discharge of preterm infants with a birth weight ≤ 1500 g and/or a gestational age ≤ 32 weeks. As previously reported, the FCC intervention was associated with a significantly lower PMA at discharge and an increased proportion of infants discharged with NGT feeding [12].

The present study analyses parental psychological outcome, specifically symptoms of anxiety and depression at discharge and at three months corrected age (CA) after discharge of very preterm infants with full oral feeding or with home NGT feeding.

Eligible participants were parents of preterm infants with a birth weight ≤ 1500 g and/or a gestational age ≤ 32 weeks, without major congenital anomalies and without a prior decision for palliative care at the time of enrolment. Exclusion criteria included severe parental psychiatric conditions, such as acute psychosis. Written informed consent was obtained from all participating parents prior to inclusion.

### 2.2. Outcome Parameters

Anxiety and depressive symptoms were assessed using the German version (HADS-D) of the Hospital Anxiety and Depression Scale (HADS). The HADS is a validated 14-item self-report questionnaire comprising two subscales assessing anxiety and depression, respectively, with higher scores indicating greater symptom burden [18]. In addition, anxiety and depression scores were dichotomized according to the prespecified threshold for clinically significant symptomatology. Scores of 8 or higher were classified as clinically significant, whereas scores below 8 were classified as not clinically significant [19].

Four weeks after discharge, parents completed a previously published self-assessment questionnaire evaluating perceived discharge readiness, feeding-related stress at home, and the timing of discharge [7]. Preparedness for discharge and feeding-related stress were rated using 5-point Likert scales, with higher scores indicating greater preparedness and higher stress levels, respectively. Assessment of the timing of discharge was classified as “too early”, “appropriate”, or “too late”. Questionnaires were completed during routine follow-up visits or returned by mail. Non-responders were contacted by telephone. For parents who were not fluent in German, questionnaires were completed with the assistance of a certified interpreter.

### 2.3. Data Collection

Clinical data on infants were extracted from hospital medical records. Baseline sociodemographic characteristics of parents were assessed using a self-administered questionnaire. Mothers and fathers independently completed the HADS-D within the week prior to discharge and again at 3 months CA during routine follow-up visits. In the case of twins or triplets, each parent completed a single questionnaire referring to all infants. If at least one of the multiples was discharged on NGT feeding, the parents were classified as NGT feeding parents.

### 2.4. Statistical Analysis

Data analysis was conducted using and R, version 4.6.1 (The R Foundation for Statistical Computing, Vienna, Austria). Data are presented as mean (standard deviation, SD), mean (standard error, SE; and 95% confidence interval, CI), or as number (percentage) as appropriate. The figures illustrate mean and 95% CI. The chi-square test was used to compare categorical variables between the groups. ANOVA was used to compare continuous variables between the groups. Mean (SD) parental HADS-D depression and anxiety scores at discharge and at 3 months CA follow-up, and the corresponding percentages of parents with clinically relevant depression or anxiety scores are presented in Supplemental Table S1, including straightforward statistical comparisons between the groups.

#### 2.4.1. Analyses of HADS-D Scores by Linear Mixed-Effects Models

Maternal and paternal anxiety and depression scores were analyzed using separate linear mixed-effects models with a random intercept for the maternal and paternal ID to account for the correlation of repeated measurements.

Each model included NGT at discharge (yes or no), assessment time (discharge or three months CA), and the NGT-by-time interaction as fixed effects.

Fixed effects were evaluated using Type III tests with Satterthwaite approximations for the denominator degrees of freedom. Estimated marginal means, standard errors, and 95% confidence intervals (CI) were calculated for each combination of NGT and assessment time, with denominator degrees of freedom approximated using the Kenward-Roger method. Pairwise contrasts, also based on the Kenward-Roger approximation, were used to compare the two NGT groups at each assessment and to evaluate changes from discharge to three months CA within each NGT group.

Because NGT-by-time interactions were evaluated for four related outcomes, the interaction *p*-values were additionally adjusted using the Bonferroni correction.

#### 2.4.2. Sensitivity Analysis of Clinically Relevant Symptom Scores

As a sensitivity analysis, separate population-averaged logistic regression models were fitted for maternal and paternal anxiety and depression scores ≥ 8 using generalized estimating equations (GEE) with a logit link function. Within-participant correlation between the repeated measurements was accounted for through an exchangeable working correlation structure, and robust (sandwich) standard errors were used for inference. The models included NGT, assessment time, and the NGT-by-time interaction as explanatory variables. The NGT-by-time interaction tested whether changes in the probability of clinically relevant symptoms differed between the two NG groups.

Marginal odds ratios and predicted probabilities with 95% CI were calculated from the fitted models.

Post hoc contrasts compared the NGT groups at each assessment and compared discharge with the three-month CA follow-up within each NGT group. Because the NGT-by-time interaction was evaluated for four outcomes, the interaction *p*-values were additionally adjusted using the Bonferroni correction.

All statistical tests were two-sided, and an unadjusted *p*-value below 0.05 was considered statistically significant.

## 3. Results

Between October 2020 and May 2024, 340 infants were eligible for study participation. A total of 102 infants (30%) were excluded, including 18 whose families declined participation, 34 who were not approached because of staff shortages, 44 who met exclusion criteria, and 6 who died before discharge. The remaining 238 infants constituted the study cohort. Of these, 131 were discharged with full oral feeding and 107 with home NGT feeding.

Baseline characteristics are presented in Table 1. Infants discharged without NGT feeding were born at a higher PMA than those discharged with NGT feeding (29.1 ± 2.58 vs. 28.1 ± 2.96 weeks, *p* < 0.001) and had a higher birth weight (1189 ± 384 g vs. 1015 ± 352 g, *p* < 0.001). At discharge, infants in the non-NGT-feeding group had a higher weight (2624 ± 480 g vs. 2432 ± 582 g, *p* < 0.001), greater length (46.97 ± 2.45 cm vs. 45.4 ± 2.6 cm, *p* < 0.001), and larger head circumference (32.8 ± 1.52 cm vs. 31.7 ± 1.68 cm, *p* < 0.001).

Retinopathy of prematurity was less common among infants discharged without NGT feeding (1.5% vs. 7.5%, *p* = 0.02), whereas no significant differences were observed for other neonatal morbidities. Sociomedical follow-up was recommended and offered to all parents, but was accepted less frequently by parents of infants discharged without NGT feeding (69.5% vs. 86.0%, *p* = 0.003).

The 238 study infants represented 201 parent–infant dyads, including 35 twin pregnancies (68 infants) and 3 triplet pregnancies (7 infants). Parental characteristics, including age, educational level, and country of origin, did not differ significantly between groups. However, these variables were not available for all parents (Table 2).

A total of 172 parent–infant dyads (86%) completed the questionnaire assessing discharge readiness and feeding-related stress (Table 3). Parental self-assessed readiness for discharge and feeding-related stress four weeks after discharge did not differ significantly between families of infants discharged with and without NGT feeding (Table 3).

At discharge, HADS-D questionnaires were completed by 155 (77%) mothers and 135 fathers (67%); at three months CA follow-up, responses were available from 121 (60%) mothers and 119 (59%) fathers (Supplemental Table S1).

At discharge, mean depression and anxiety scores and the percentages of parents with clinically significant scores did not differ significantly between parents of infants discharged with and without NGT feeding (Supplemental Table S1). However, at 3 months CA follow-up, mean depression scores in both parents, mean anxiety score in mothers, and the percentage of mothers with clinically relevant depression scores appeared to be significantly lower in the NGT feeding group. After adjusting the *p*-values for 16 comparisons (Bonferroni correction) only the lower depression score in mothers at 3 months CA follow-up remained significant (*p* = 0.032).

### 3.1. HADS-D Scores Analyses Using Linear Mixed-Effects Models

In linear mixed-effects models, maternal anxiety decreased significantly from discharge to 3 months (*p* = 0.005), and paternal depression increased over time (*p* = 0.045). No main effect of NGT reached significance. The time × NGT interaction was nominally significant for maternal depression (*p* = 0.017) and paternal anxiety (*p* = 0.032) but not after Bonferroni correction (corrected *p* = 0.067 and 0.126). The interactions for maternal anxiety (*p* = 0.130) and paternal depression (*p* = 0.114) were non-significant throughout (Table 4).

**Figure 1:**
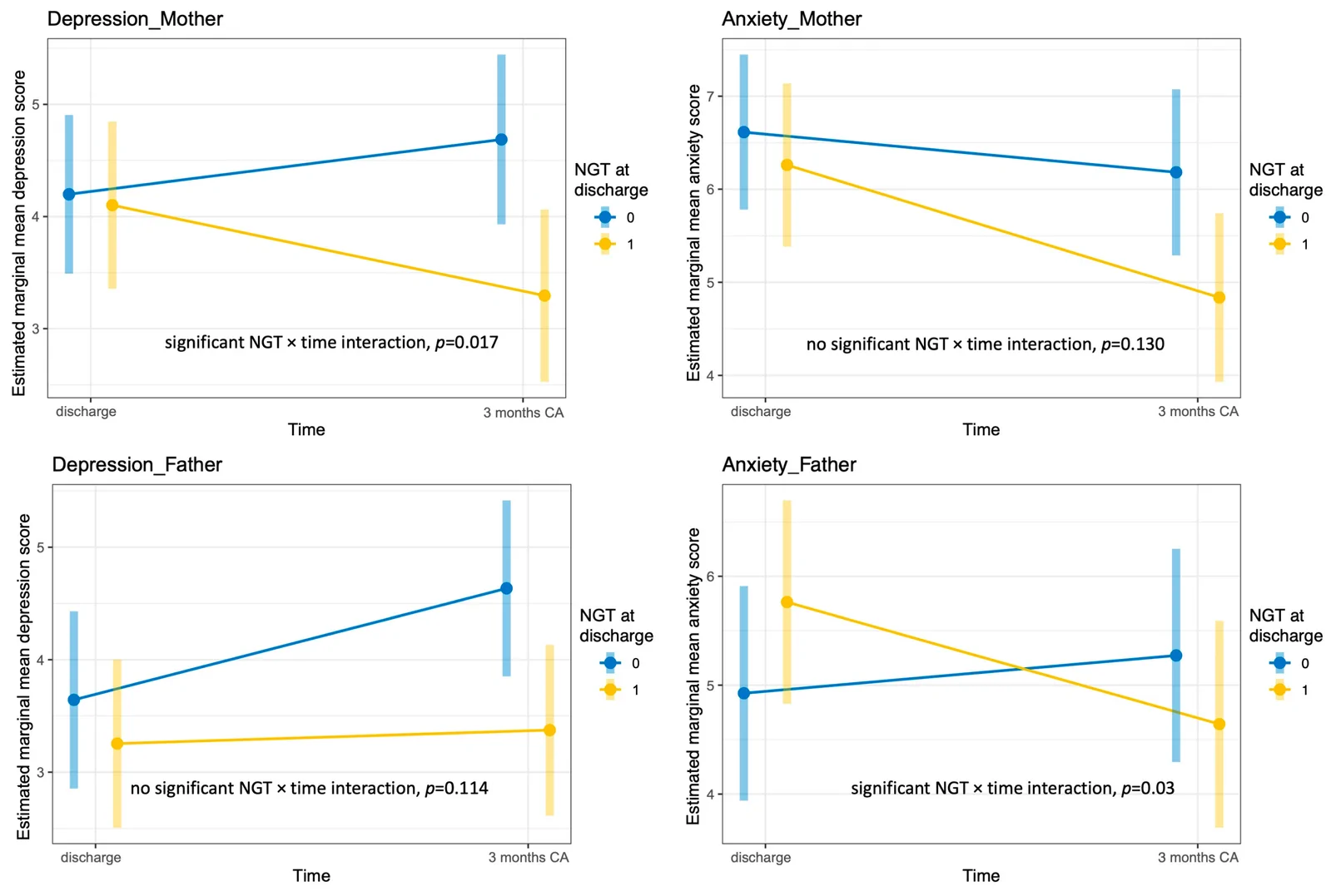
Estimated marginal means of parental anxiety and depression across the post-discharge course, by nasogastric tube (NGT) feeding status at discharge. Panels show HADS anxiety (HADS-A) and depression (HADS-D) scores for mothers and fathers at discharge and at the 3-month corrected-age (CA) follow-up. Points are estimated marginal means from linear mixed-effects models (fixed effects: NGT feeding, time, and their interaction as well as a random intercept per participant), and error bars denote 95% confidence intervals. Blue lines indicate infants discharged without NGT, yellow lines those discharged with NGT.

Across outcomes, the trajectories were consistent: parental distress tended to decline more in the NGT group, most visibly for maternal and paternal anxiety and maternal depression, while paternal depression rose over time in the non-NGT group. As no interaction survived correction, these group differences in trajectory are exploratory and are shown in Figure 1 and Supplemental Table S2.

### 3.2. Sensitivity Analysis of Clinically Relevant Symptom Scores

In population-averaged logistic GEE models, the probability of a clinically relevant maternal anxiety score decreased significantly from discharge to 3 months (*p* = 0.036), as did clinically relevant paternal anxiety score (*p* = 0.020). No main effect of NGT status reached statistical significance. The time × NGT interactions were nonsignificant for all models (Table 5).

**Figure 2:**
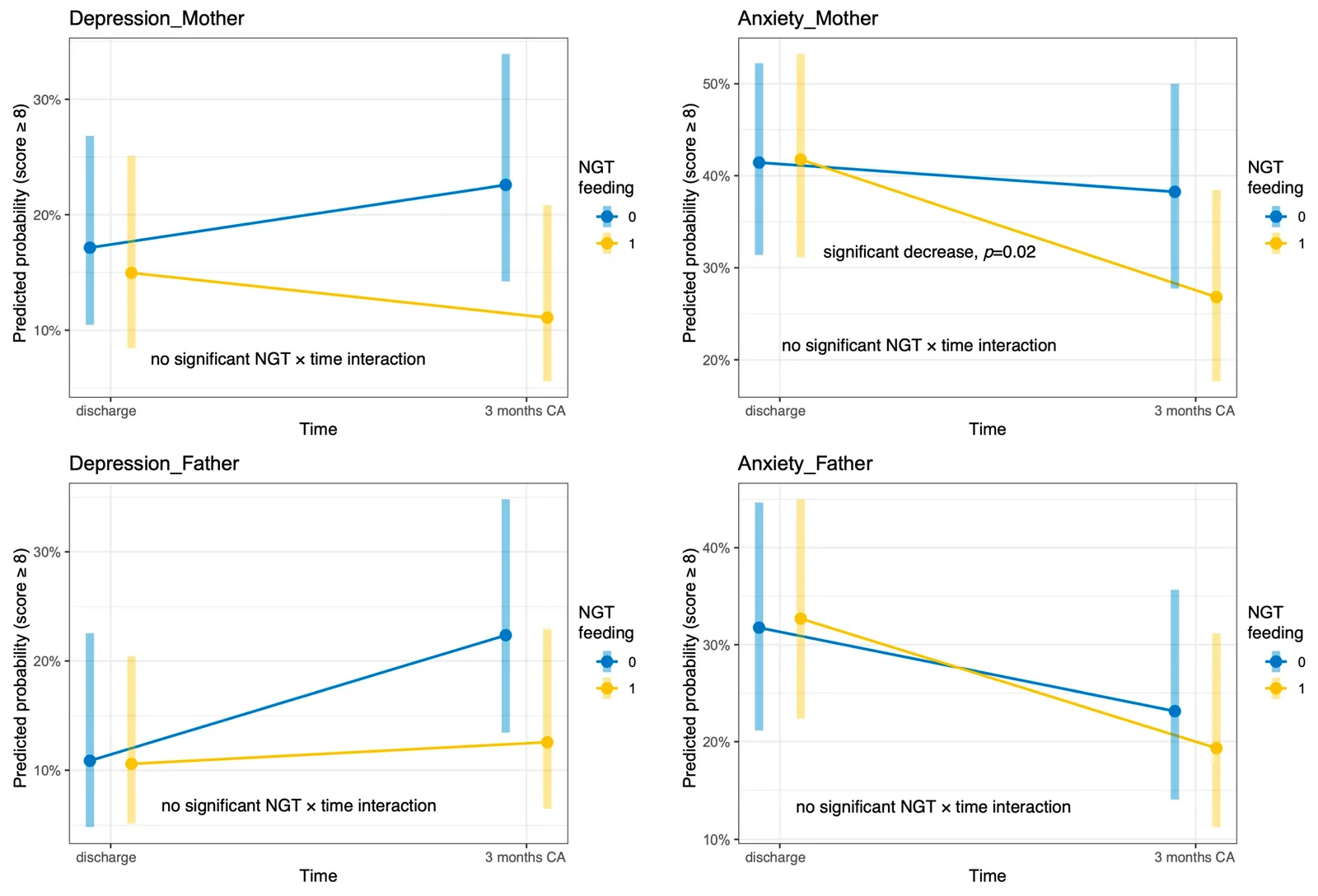
Estimated probabilities of clinically significant HADS-D depression and anxiety scores (≥8) in parents at discharge and at three months CA follow-up based on logistic generalized estimated equations. Panels show percentage of parents with clinically significant HADS-D anxiety and depression scores (≥8) at discharge and at 3-month corrected-age (CA) follow-up. Points are estimated marginal probabilities from population-averaged logistic regression models (fixed effects: NGT at discharge, time, and their interaction as well as a random intercept per participant), and error bars denote 95% confidence intervals. Blue lines indicate parents of infants discharged without NGT, yellow lines with NGT.

Across outcomes, the descriptive trajectories generally suggested a lower parental probability for clinically significant distress in the NGT group, particularly for clinically significant maternal anxiety and depression. The probability for clinically significant parental anxiety decreased in both groups, whereas the probability for clinically significant parental depression increased more clearly in the non-NGT group. Within the NGT group, the reduction in the probability for clinically significant maternal anxiety was statistically significant (*p* = 0.023), while the reduction in the probability for clinically significant paternal anxiety approached significance (*p* = 0.051). However, because none of the interaction tests was significant, these apparent between-group differences in trajectories should be interpreted as exploratory (Figure 2 and Supplemental Table S3).

## 4. Discussion

To our knowledge, this is the first study to evaluate symptoms of depression and anxiety in both mothers and fathers at discharge and at three months CA following early discharge with NGT feeding in very preterm infants.

Infants discharged with NGT feeding were born at a significantly lower gestational age than infants discharged on full oral feeding. However, PMA at discharge did not differ between the groups. In general, PMA at discharge is inversely associated with PMA at birth, infants born at lower gestational ages are typically discharged at a higher PMA [20]. Therefore, the finding that more immature infants were discharged at a similar PMA suggests that home NGT feeding may facilitate earlier discharge and reduce prolonged hospitalization in this particularly vulnerable population. However, evidence regarding the impact of home NGT feeding on length of hospital stay remains scarce. Available studies, consistent with our findings, have reported shorter hospital stays among infants discharged with home NGT feeding [6,13].

The principal finding of our study is that early discharge with home NGT feeding was not associated with increased symptoms of anxiety or depression in either mothers or fathers at discharge. At three months CA, parents of infants discharged with home NGT feeding reported similar or more favorable mental health outcomes than parents of infants discharged on full oral feeding. Overall, these findings suggest that early discharge with home NGT feeding is not associated with increased parental psychological distress during the transition from hospital to home.

Compared with population-based German reference data, mean depression scores among females and males were similar, whereas mean anxiety scores were elevated. The prevalence of clinically significant anxiety symptoms (HADS-D score ≥ 8) was higher than in the general population, while the prevalence of clinically significant depressive symptoms (HADS-D score ≥ 8) was comparable to that reported in a population-based sample [21].

Previous studies evaluating parental psychological outcome following early discharge have reported comparable findings. In a cohort of 140 more mature preterm infants (median PMA at birth 32–33 weeks), early discharge without tube feeding was not associated with increased parental stress, anxiety, or depression at discharge, despite lower discharge weights and shorter hospital stays. Mothers in the early discharge group reported lower depressive symptom scores at discharge [22]. Similarly, among 75 more mature preterm infants (mean PMA at birth 31–32 weeks) discharged with NGT feeding and domiciliary nursing support, maternal anxiety was lower at discharge, whereas paternal anxiety did not differ between groups [9].

In the present study, infants were significantly less mature at birth than those included in previous studies. Nevertheless, early discharge with home tube feeding was not associated with increased parental anxiety or depression at discharge. In contrast to previous reports, however, no reduction in parental symptoms was observed at the time of discharge [9,22].

The linear mixed models suggested significant positive trends in maternal depression scores and paternal anxiety scores at 3 months CA follow-up with early discharge with NGT feeding. Significant NGT-by-time interactions were observed. Neither interaction remained statistically significant after adjustment for multiple testing. The sensitivity analyses based on clinically significant symptom thresholds did not provide evidence that changes over time differed significantly between the two NGT groups. Although several within-group changes reached nominal statistical significance, these findings cannot be interpreted as evidence of differential treatment or group effects in the absence of significant NGT-by-time interactions. However, categorizing a continuous score discards information, reduces statistical power. Nevertheless, early discharge with home tube feeding was not associated with increased parental anxiety or depression at 3 months CA discharge.

One potential explanation for the observed positive trends with early discharge on NGT feeding may be that discharge was implemented within a comprehensive family-centered care (FCC) framework. Following FCC implementation, home NGT feeding became standard practice and was introduced to families early on during the hospital stay. Parents were actively involved as primary caregivers and integral members of the care team, including participation in medical rounds and discharge planning. Training in NGT feeding started within the first week of life, allowing parents to gradually build competence and confidence. In addition, structured parental support groups and sociomedical follow-up after discharge were provided. This continuous preparation and empowerment were reflected in high levels of perceived discharge readiness. FCC has consistently been associated with improved parental psychological outcomes, including reduced anxiety, depression, and stress among parents of preterm infants [23,24]. These benefits have primarily been demonstrated at the time of discharge; however, infants in those studies were discharged without tube feeding. For example, a study from the Netherlands reported lower stress levels at discharge among parents of preterm infants (median PMA 32–34 weeks) managed within a FCC model compared with standard care [25,26]. Similarly, a large cluster-randomized trial from China including preterm infants born at 28–35 weeks of gestation found significantly lower maternal stress levels at discharge in the FCC group [27].

Importantly, healthcare professionals may traditionally perceive discharge without NGT feeding as less burdensome for parents compared with discharge with ongoing NGT feeding at home. However, the emotional impact of being discharged home earlier and the associated increase in parental confidence and sense of competence in caring for their own infant may outweigh the perceived additional burden of tube feeding. Moreover, improved access to social support within the family at home may facilitate parental self-care and, consequently, promote better parental mental health. Despite the additional responsibilities associated with NGT feeding, including feeding management and tube care, parents in our cohort did not report higher levels of depression or anxiety at discharge. Notably, infants discharged with tube feeding on average had lower PMA and birth weight and experienced greater neonatal morbidity, reflecting a more medically complex population than those included in previous FCC studies. Nevertheless, active parental involvement in the care of these highly vulnerable infants was not associated with increased psychological burden and may have contributed to the favorable parental outcomes observed during follow-up.

This interpretation is supported by large surveys suggesting that positive parental experiences with FCC during the NICU stay are associated with fewer depressive symptoms both at discharge and at four months PMA [23,28]. Similarly, the more favorable psychological outcomes observed in our cohort persisted beyond discharge. Taken together, these findings suggest that early discharge of more medically complex preterm infants may be feasible at least within a structured FCC framework.

Finally, discharge readiness was perceived high in our study population. Within the framework of FCC, parents were actively involved in their infant’s care from an early stage of the NICU stay as primary caregivers, which may have enhanced their confidence and competence in caring for their infant. In addition, early training in NGT management may have enabled parents to safely assume tube feeding responsibilities at home and may have contributed to the high discharge readiness observed in our cohort. These findings are consistent with previous studies demonstrating improved discharge readiness, including greater confidence in infant feeding, assessment of the infant’s general condition, and hygienic care, among parents of preterm infants participating in FCC programs [23]. Thus, FCC may represent an important framework supporting the successful implementation of early discharge strategies, including home NGT feeding, although its individual contribution cannot be separated from the effects of the discharge approach itself in this observational study.

As expected, there was a trend towards more feeding-related stress (*p* = 0.07) among parents of infants discharged with tube feeding, suggesting that additional support and education regarding feeding and tube management at home may further improve the transition from hospital to home.

Nearly all the parents whose infants were discharged with NGT feeding decided to have sociomedical follow-up (86%), which was offered to all parents equally, free of charge. The joint decision to discharge with NGT feeding by the medical team and the parents was made upfront independently of later sociomedical follow-up. In fact, the parents arranged all but the first outpatient socio-medical follow-up appointments with the follow-up team after discharge. It may be speculated that sociomedical follow-up may have mediated potential NGT effects, but it is beyond the scope of the present paper to separate direct NGT and mediated NGT effects because of the complexity of the required models and the limited sample size.

This study has several limitations. First, as a single-center study, the generalizability of our findings to other neonatal units may be limited. Second, the observational study design precludes causal inference, and residual confounding cannot be excluded. Randomization in providing the option of early discharge with NGT feeding or not would have been required. Third, HADS-D questionnaire participation was 77% (*n* = 155) among mothers and 67% (*n* = 135) among fathers at discharge and declined further by three months CA (Table 3), which may have introduced response bias as parents with developed depression and anxiety symptoms might be less prepared to respond. Moreover, parental symptoms of depression and anxiety were not adjusted for baseline mental health status e.g., HADS score assessed before or at admission. Therefore, pre-existing differences between the groups may have introduced bias into our findings. Finally, the present study with about one hundred parent infant dyads in each group is still underpowered to justify robust hypothesis on at least 4 distinct HADS outcomes (depression and anxiety scores in mothers and fathers) which required adjustment for multiple testing. Despite these limitations, to our knowledge, this is the first study to evaluate parental outcomes associated with early discharge with home NGT feeding in very preterm infants. Future multicenter studies are needed to confirm our findings and assess their generalizability across different clinical settings.

## 5. Conclusions

Discharge of very preterm infants with home NGT feeding was not associated with increased maternal or paternal symptoms of anxiety or depression at discharge and at 3 months CA compared with discharge on full oral feeding. Across the outcomes, parental distress tended to be lower at 3 months CA with NGT feeding at discharge. However, as no NGT × time interaction survived correction for multiple testing, these group differences should be interpreted as exploratory in nature. Furthermore, high levels of parental discharge readiness were observed in families discharged with home NGT feeding. While previous studies have primarily demonstrated benefits for infants, these findings suggest that home NGT feeding may also be a feasible and well-supported approach from a parental perspective, without evidence of increased psychological burden.

## Figures and Tables

**Table 1:** Infant characteristics and neonatal morbidities.

	No NGT at Discharge	NGT at Discharge	*p* (ANOVA or χ^2^-Test)
N	131	107	
Cesarean section, *n* (%)	125 (95.42)	106 (99.07)	ns
Male sex, *n* (%)	62 (47.33)	58 (54.21)	ns
Birthweight, g (SD)	1189 (384)	1015 (352)	<0.001
PMA at birth, weeks (SD)	29.09 (2.58)	28.1 (2.96)	<0.001
Weight at discharge, g (SD)	2624 (480)	2432 (582)	<0.001
Length, cm (SD)	47.0 (2.45)	45.4 (3.17)	<0.001
Head circumference, cm (SD)	32.8 (1.52)	31.7 (1.68)	<0.001
PMA at discharge, weeks (SD)	37.19 (2.45)	36.86 (2.86)	0.34
BPD, *n* (%)	12 (9.2%)	10 (9.4%)	0.96
IVH ≥ 3, *n* (%)	2 (1.5%)	6 (5.6%)	0.08
PVL, *n* (%)	2 (1.5%)	1 (0.9%)	0.67
ROP ≥ 3, *n* (%)	2 (1.54%)	8 (7.5%)	0.02
NEC ≥ 2, *n* (%)	3 (2.3%)	1 (0.9%)	0.42
FIP, *n* (%)	4 (3.1%)	3 (2.8%)	0.91
Sociomedical follow-up, *n* (%)	91 (69.5%)	92 (86.0%)	0.003

Data given as mean (SD = standard deviation) or numbers and percentages; PMA = Postmenstrual Age); BPD = Bronchopulmonary Dysplasia; IVH = Intraventricular Haemorrhage, PVL = Periventricular Leucomalacia, ROP = Retinopathy of Prematurity, NEC = Necrotizing Enterocolitis, FIP = Focal Intestinal Perforation, NGT = Nasogastric Tube, ns = not significant.

**Table 2:** Characteristics of Mothers and Fathers.

	No NGT at Discharge	NGT at Discharge	*p* (ANOVA or χ^2^-Test)
Discharged parent-infant dyads	106	95	
Mothers			
Age, *n*	81	73	
Age, years, mean (+/−SD)	32.6 (5.3)	31.9 (6.1)	0.42
Country of origin, *n*	64	66	
German, *n* (%)	40 (62%)	46 (70%)	0.39
Non-German, *n* (%)	24 (38%)	20 (30%)	
Level of education, *n*	85	72	
No High School, *n* (%)	35 (41%)	33 (46%)	0.14
High School, *n* (%)	18 (21%)	22 (30%)	
University Degree, *n* (%)	32 (38%)	17 (24%)	
Fathers			
Age, *n*	59	59	
Age, years, mean (+/−SD)	34.7 (5.7)	35.2 (5.8)	0.66
Country of origin, *n*	46	48	
German, *n* (%)	32 (70%)	40 (83%)	0.12
Non-German, *n* (%)	14 (30%)	8 (17%)	
Education level, *n*	62	58	
No High School, *n* (%)	26 (42%)	24 (41%)	0.9
High School, *n* (%)	15 (24%)	16 (28%)	
University Degree, *n* (%)	21 (34%)	18 (31%)	

Data given as mean (SD = standard deviation) or number and percentage.

**Table 3:** Parental Self-Assessment after Discharge.

	No NGT at Discharge	NGT at Discharge	*p* (χ^2^-Test)
Parents, *n*	106	95	
Responding parents, *n* (%)	86 (81%)	86 (91%)	0.06
Stress with feeding 4–5, *n* (%)	16 (15.1%)	25 (26.3%)	0.11
Discharge Readiness 4–5, *n* (%)	72 (83.7%)	76 (88.4%)	0.38
Time of discharge (too early/right/too late), *n* (%)	6/70/9 (7%/82%/11%)	8/72/6 (69%/84%/7%)	0.64

Data given as number (percentage).

**Table 4:** Effects of nasogastric (NGT) feeding, time, and their interaction on parental anxiety and depression, from linear mixed models.

Outcome	Effect	F	*df*	*p*	*p* (Bonferroni)	η^2^
Anxiety, mother	NGT	2.49	1168.7	0.117	-	0.01
	Time	8.10	1135.9	0.005	-	0.06
	NGT × Time	2.32	1135.9	0.130	0.522	0.02
Depression, mother	NGT	2.59	1166.5	0.109	-	0.02
	Time	0.35	1131.5	0.553	-	0.00
	NGT × Time	5.86	1131.5	0.017	0.067	0.04
Anxiety, father	NGT	0.03	1138,9	0.864	-	0.00
	Time	1.32	1109.9	0.254	-	0.01
	NGT × Time	4.74	1109.9	0.032	0.126	0.04
Depression, father	NGT	2.97	1144.7	0.087	-	0.02
	Time	4.11	1116.6	0.045	-	0.03
	NGT × Time	2.54	1116.6	0.114	0.456	0.02

For each outcome (HADS anxiety and depression subscales in mothers and fathers), the table reports the between-subjects effect of nasogastric tube (NGT) feeding, the within-subjects effect of time (discharge vs. 3-month corrected-age follow-up), and the NGT × time interaction. Fixed effects were tested with Type III F-tests using Satterthwaite degrees of freedom; partial η^2^ is reported as the effect size. To control the false-positive rate across the four outcomes, the interaction *p*-values were Bonferroni-corrected.

**Table 5:** Estimated probabilities of clinically relevant HADS-D depression and anxiety scores (≥8) in parents at discharge and at three months CA follow-up based on logistic generalized estimated equations.

Outcome	Effect	Odds Ratio	Wald Statistic	*p*	*p* (Bonferroni)
Anxiety, mother	NGT	1.14	0.80	0.371	-
	Time	1.22	4.42	0.036	-
	NGT × Time	0.87	1.99	0.158	0.633
Depression, mother	NGT	1.28	1.96	0.161	-
	Time	1.00	0.00	0.997	-
	NGT × Time	0.842	1.51	0.220	0.878
Anxiety, father	NGT	1.05	0.075	0.784	-
	Time	1.33	5.42	0.020	-
	NGT × Time	0.935	0.305	0.581	>0.999
Depression, father	NGT	1.20	0.63	0.428	-
	Time	0.77	3.34	0.068	-
	NGT × Time	0.847	1.33	0.249	0.996

For each outcome (HADS-D anxiety and depression subscales in mothers and fathers), the table reports the between-subjects effect of nasogastric tube (NGT) feeding, the within-subjects effect of time (discharge vs. 3-month CA follow-up), and the NGT × time interaction. Effects were tested using Wald test. To control the false-positive rate across the four outcomes, the interaction *p*-values were Bonferroni-corrected.

## Data Availability

The raw data supporting the conclusions of this article will be made available by the authors on request. For privacy reason the data are not public.

## Supplementary Materials

The following supporting information can be downloaded at: https://www.mdpi.com/article/10.3390/nu18162728/s1. Table S1: HADS-D Depression and Anxiety Scores in Mothers and Fathers at discharge and at three months CA follow-up; Table S2: Estimated marginal means (standard error, SE) with 95% confidence intervals of parental anxiety and depression scores at discharge and 3-month corrected-age follow-up, by nasogastric tube feeding status, from linear mixed models; Table S3: Population-averaged estimated probabilities of clinically relevant parental anxiety and depression at discharge and 3-month corrected-age follow-up, by nasogastric tube feeding status, from logistic generalized estimating equation models.

## Abbreviations

The following abbreviations are used in this manuscript:

BPD	Bronchopulmonary Dysplasia
CA	Corrected Age
FCC	Family Centered Care
FIP	Focal Intestinal Perforation
GEE	Generalized Estimating Equations, German Version
HADS-D	Hospital Anxiety and Depression Score, German Version
IVH	Intraventricular Hemorrhage
NEC	Necrotizing Enterocolitis
NICU	Neonatal Intensive Care Unit
NGT	Nasogastric tube
PMA	Postmenstrual Age
SD	Standeard Deviation
ROP	Retinopathy of Prematurity
