# Supplementary material for: Early Discharge of Very Preterm Infants with Home Nasogastric Tube Feeding Is Not Associated with Increased Parental HADS Anxiety or Depression Scores
Source: Nutrients. 2026 Aug 20;18(16):2728. doi: 10.3390/nu18162728 (PMC13516564; doi:10.3390/nu18162728)
Supplement: Supplementary file 1 [file nutrients-18-02728-s001.zip › nutrients-4435502-supplementary.pdf]

Supplemental Table S1. HADS-D Depression and Anxiety Scores in Mothers and Fathers at discharge and at three months CA follow-up

		No NGT at discharge	NGT at discharge	p (ANOVA or χ^2-test)
	N	106	95	
**HADS-D mother (Depression score)**				
At discharge	n	82 (77.36%)	73(76.84%)	
	Mean (SD)	4.14 (3.29)	4.15 (3.27)	0.99
	N (%) ≥8	14 (17.07%)	11 (15.07%)	0.73
At 3 months CA	n	68 (64.15%)	67 (70.53%)	
	Mean (SD)	4.95 (3.62)	3.18 (2.89)	0.01
	N (%) ≥8	16 (23.53%)	7 (10.45%)	0.04
**HADS-D father (Depression score)**				
At discharge	n	57 (53.77%)	64 (67.37%)	
	Mean (SD)	3.68 (2.84)	3.27 (2.97)	0.48
	N (%) ≥8	7 (12.28%)	7 (10.94%)	0.93
At 3 months CA	n	58 (54.71%)	61 (64.21%)	
	Mean (SD)	4.78 (3.74)	3.48 (2.72)	0.04
	N (%) ≥8	14 (24.14%)	8 (13.11%)	0.12
**HADS-D mother (Anxiety score)**				
At discharge	n	82 (77.36%)	73 (76.84%)	
	Mean (SD)	6.68 (4.15)	6.34 (3.69)	0.59
	N (%) ≥8	34 (41.46%)	31 (42.47%)	0.90
At 3 months CA	n	68 (64.15%)	67 (70.53%)	0.34
	Mean (SD)	6.42 (3.85)	4.79 (3.72)	0.01
	N (%) ≥8	28 (41.18%)	18 (26.87%)	0.08
**HADS-D father (Anxiety score)**				
At discharge	n	57 (53.55%)	64 (64.21%)	
	Mean (SD)	4.88 (3.99)	5.95 (3.97)	0.14
	N (%) ≥8	18 (31.58%)	22 (34.34%)	0.74
At 3 months CA	n	58 (54.71%)	61 (64.21%)	0.17
	Mean (SD)	5.46 (3.81)	4.74 (3.75)	0.29
	N (%) ≥8	14 (24.14%)	12 (19.67%)	0.56

Data given as mean (SD = standard deviation) or numbers (percentage).
HADS-D = Hospital Anxiety and Depression Score, German Version, PMA = Postmenstrual Age;

Supplemental Table S2. Estimated marginal means (standard error, SE) with 95% confidence intervals of parental anxiety and depression scores at discharge and 3-month corrected-age follow-up, by nasogastric tube feeding status, from linear mixed models.

Score / time point	No NGT	NGT
**HADS-D, mother (anxiety)**		
At discharge	6.61 (0.42) [5.78, 7.45]	6.26 (0.45) [5.38, 7.14]
At 3 months CA	6.18 (0.45) [5.29, 7.08]	4.84 (0.46) [3.93, 5.74]
**HADS-D, mother (depression)**		
At discharge	4.20 (0.36) [3.49, 4.91]	4.10 (0.38) [3.36, 4.85]
At 3 months CA	4.69 (0.38) [3.93, 5.44]	3.29 (0.39) [2.53, 4.06]
**HADS-D, father (anxiety)**		
At discharge	4.93 (0.50) [3.94, 5.91]	5.76 (0.47) [4.83, 6.70]
At 3 months CA	5.27 (0.50) [4.29, 6.25]	4.64 (0.48) [3.69, 5.59]
**HADS-D, father (depression)**		
At discharge	3.64 (0.40) [2.85, 4.43]	3.25 (0.38) [2.51, 4.00]
At 3 months CA	4.63 (0.40) [3.85, 5.42]	3.37 (0.39) [2.61, 4.13]

Values are estimated marginal means (SE) [95% CI] derived from linear mixed-effects models (fixed effects: NGT feeding, time, and their interaction; random intercept per participant).

Abbreviations: HADS-D - Hospital Anxiety and Depression Scale, anxiety/depression subscale (German version); **NGT** - nasogastric tube; **CA** - corrected age; **SE** - standard error; **CI** - confidence interval

Supplemental Table S3. Population-averaged estimated probabilities of clinically relevant parental anxiety and depression at discharge and 3-month corrected-age follow-up, by nasogastric tube feeding status, from logistic generalized estimating equation models.

**Outcome / time point**	**No NGT**	**NGT**
**HADS-D, mother (clinically relevant anxiety)**		
At discharge	41.4% (5.4%) [31.4%, 52.2%]	41.8% (5.7%) [31.1%, 53.2%]
At 3 months CA	38.3% (5.8%) [27.7%, 50.0%]	26.8% (5.3%) [17.7%, 38.5%]
**HADS-D, mother (clinically relevant depression)**		
At discharge	17.1% (4.1%) [10.5%, 26.8%]	15.0% (4.2%) [8.5%, 25.1%]
At 3 months CA	22.6% (5.0%) [14.2%, 33.9%]	11.1% (3.7%) [5.6%, 20.8%]
**HADS-D, father (clinically relevant anxiety)**		
At discharge	31.8% (6.1%) [21.2%, 44.7%]	32.7% (5.8%) [22.4%, 45.0%]
At 3 months CA	23.1% (5.5%) [14.1%, 35.7%]	19.3% (5.0%) [11.3%, 31.2%]
**HADS-D, father (clinically relevant depression)**		
At discharge	10.9% (4.3%) [4.9%, 22.6%]	10.6% (3.7%) [5.2%, 20.4%]
At 3 months CA	22.4% (5.5%) [13.5%, 34.8%]	12.6% (4.1%) [6.5%, 22.9%]

Values are population-averaged estimated probabilities (SE) [95% CI] derived from logistic GEE models with a binomial distribution and logit link. The models included NGT feeding status at discharge, time, and their interaction and accounted for repeated measurements within participants. Clinically relevant symptoms were defined as HADS scores ≥8.

Abbreviations: HADS-D, Hospital Anxiety and Depression Scale anxiety/depression subscale, German version; NGT, nasogastric tube; CA, corrected age; SE, standard error; CI, confidence interval; GEE, generalized estimating equation.
